# Post-Graduate Study in Medicine.—II

**Published:** 1912-06-15

**Authors:** 


### POST-GRADUATE STUDY IN MEDICINE.—II.*

### The Continental and English Systems of Study Compared.

The English graduate who becomes acquainted with the previously mentioned courses can only envy his German colleague the opportunities and facilities which they afford the latter. The question remains whether he cannot try to imitate the German example and bring about in England a similar organisation, a similar bureau, a similar Central Committee, and similar facilities. To some extent the Polyclinic in Chenies Street, which is a private undertaking receiving no State support, does the work of a central bureau, while the various post-graduate schools already form the nucleus of post-graduate academies on the German model.

The great objection to our existing schools are that they are not available for the practitioner who really deserves a helping hand. They appeal to metropolitan and suburban doctors, not to those in country districts; their fees are in the majority of cases very high, and in none of them gratuitous teaching is provided. So long as the schools are poor, living hand to mouth, and unassisted by the State, it would be unreasonable to expect free classes, but the promotion of graduate study in medicine and dentistry is, from a national point of view, a matter of such primary importance that it is impossible for England persistently to lag behind Germany and Russia in dealing with it. A large number of general practitioners will avail themselves of opportunities to perfect their knowledge and to keep abreast of the latest developments in medical art, so long as such opportunities are given upon lines somewhat similar to those that exist in Germany. There are many difficulties in the way of attaining to that success which has crowned the efforts of the *Central Komitee für das aerztliche Fortbildungswesen*, but they are not insuperable. A certain amount of jealousy and rivalry between existing schools will doubtless be the most serious obstacle in the way of obtaining unity of ideal and unanimity of action. For example, the desirability of having a central bureau which may serve as general headquarters of the movement in this country seems obvious, yet that such a central institution should be evolved out of any already existing school or clinic is scarcely to be expected or desired. A movement in which the whole Empire should participate is not to be made to serve as a splendid advertisement for any particular institution or school, and it is equally undesirable that it should be centralised in a university town, where the student teaching is bound to clash with the graduate instruction. Properly such a movement should be metropolitan.

It should have its headquarters in London, with branches all over the country, either in the shape of permanent courses or as temporary classes, held at times and places determined by the wishes of country practitioners who may desire to attend them. The headquarters institution need not entail a colossal or extraordinary expense. The Kaiserin Friedrich Haus was built and equipped at a cost of less than £60,000, and is to some extent self-supporting, owing to the fact that exhibition stalls are let out at a yearly rental to certain firms. Once the public is roused to the fact that the merits of such an institution and such a movement are not parochial, but that an efficient and thoroughly competent medical service is imperatively necessary in the interests of the nation, the question of State aid will no longer be a difficult one. It will be recognised that the small annual grant, supported if need be by donations from the Colonies—since they will benefit as much as the Mother Country by the establishment of such an institution—will be sufficient to augment the income derived from the leases of exhibition stalls to make the institution self-supporting. There is no necessity to lay stress on the educative value of a permanent exhibition, such as exists in the Kaiserin Friedrich Haus, and should exist in the London institution if the latter ever comes into being. On few points does the public need constant and continual instruction so much as in matters of social hygiene, medical sociology, and the many ancillary subjects which profoundly stir popular imagination because of the obscurity that surrounds them. By serving as a semi-popular centre, where lectures can be given, where a permanent exhibition can be visited daily by whomsoever desires practical instruction on some subject in the debatable land where medico-professional and popular interests are one, and, above all, by providing a visible object-lesson in solidarity and a sign of the alliance between the profession and the State, such a central institution can render valuable services to the nation no less than to medicine.

The main persons concerned in the development of graduate study are the medical practitioners themselves; from them must come the initiative, as it came in Germany and America. It is, therefore, highly gratifying to observe that there is at the present moment a possibility that the profession may be stirred into active interest in this matter of post-graduate study. For this stirring-up we have to thank Professor Kuttner, who, on the occasion of the sessional meeting of the International Medical Congress at Budapest, summoned a meeting of those interested in graduate study, delivered an inspiriting address on the present status of the movement, and obtained the formation of an International Committee for Post-Graduate Instruction in Medicine. Representatives to this Committee, the object of which is to organise graduate instruction on an international basis, are so far nominated by the respective Governments of the countries who have responded to the invitation. The British Government has nominated five members—one for England, one for Scotland, one for the Indian Medical Service, and one each for the Army and Naval Medical Services. It is true none of these gentlemen are very actively associated with graduate study in this country, nor

* The previous article appeared on June 1.

have they taken an outstanding interest in the movement, but the International Committee has the power to co-opt members, and will doubtless remedy the deficiency by co-opting to its executive body prominent leaders of the graduate-study movement in this country who are actively associated with post-graduate schools. The International Committee starts its work under promising conditions. It receives a small grant from each participating Government, and its business will be largely organising work. The value of such work at the present moment, when there is practically, outside Germany and Russia, no organisation in the movement, it is not easy to over-estimate, and the International Committee will do even more good, so far at least as this country is concerned, by prominently directing public attention to a matter that is urgently in need of public consideration.

There are many questions which demand the earnest attention of the profession, and it is highly probable that within the next few years the number of problems that stand in need of speedy solution will have increased. Few questions, however, are so important, because of their intimate relation to the welfare of the public and *ipso facto* to the health of the public, as this of the education of the practitioner. It is a matter in which the laity may well interest themselves. The profession, as such, is poor, and the financial difficulty is likely to frighten the majority of its members into inactivity. There can be no doubt that, once this difficulty is past and once the matter has been taken into the hands of an earnest, active minded Committee, a movement to endow London with an institution as fine, as representative, and as efficient as that at Berlin should prove a success.

